# Microbiome Profile of Deep Endometriosis Patients: Comparison of Vaginal Fluid, Endometrium and Lesion

**DOI:** 10.3390/diagnostics10030163

**Published:** 2020-03-17

**Authors:** Camila Hernandes, Paola Silveira, Aline Fernanda Rodrigues Sereia, Ana Paula Christoff, Helen Mendes, Luiz Felipe Valter de Oliveira, Sergio Podgaec

**Affiliations:** 1 Hospital Israelita Albert Einstein, Av. Albert Einstein 627, Morumbi, São Paulo 05651-901, Brazil; helen2002@me.com (H.M.); sergiopodgaec@me.com (S.P.); 2 BiomeHub, Av. Luiz Boiteux Piazza, 1302, Canasvieiras, Florianópolis 88056-000, Brazilaline@biome-hub.com (A.F.R.S.);

**Keywords:** vaginal fluid, microbiome, next generation sequencing (NGS), pathogenesis, endometriosis, 16S rRNA

## Abstract

This work aimed to identify and compare the bacterial patterns present in endometriotic lesions, eutopic endometrium and vaginal fluid from endometriosis patients with those found in the vaginal fluid and eutopic endometrium of control patients. Vaginal fluid, eutopic endometrium and endometriotic lesions were collected. DNA was extracted and the samples were analyzed to identify microbiome by high-throughput DNA sequencing of the 16S rRNA marker gene. Amplicon sequencing from vaginal fluid, eutopic endometrium and endometriotic lesion resulted in similar profiles of microorganisms, composed most abundantly by the genus *Lactobacillus, Gardnerella, Streptococcus* and *Prevotella*. No significant differences were found in the diversity analysis of microbiome profiles between control and endometriotic patients; however deep endometriotic lesions seems to present different bacterial composition, less predominant of *Lactobacillus* and with more abundant *Alishewanella, Enterococcus* and *Pseudomonas.*

## 1. Introduction

Endometriosis is a gynecological disorder affecting 10% to 15% of women in reproductive age; up to 70% of the patients have pelvic pain and 48% have fertility problems [1,2]. The disease is characterized by the presence of stromal and/or endometrial glandular epithelium outside the uterus and is classified in three different types: superficial, ovarian and deep endometriosis [3,4,5]. Endometriosis was first described in the seventeenth century and, despite all research efforts in the last 30 years, its pathogenesis is still unclear [6].

Nowadays the most accepted hypothesis is that the foci of endometriosis originate from retrograde menstruation [7]. According to this theory, initially proposed by Sampson, the retrograde tubal flow seeds menstrual endometrial tissue in the peritoneal cavity and other organs, to which it adheres. However, as around 90% of women have retrograde menstruation, and just only 10% develop the disease, several authors have been proposed that other factors like anatomical, genetic, endocrine, environmental and inflammatory may influence this tissue implantation [8,9,10,11,12,13,14,15,16].

One of the most described are the inflammatory factors. Several studies have already demonstrated, for example, that the peritoneal fluid of women with endometriosis has high levels of pro-inflammatory cytokines and growth factors, such as TNF-α, IL-1 and IL-6. In previous studies carried out by our research group, we described the role of inflammatory factors presented in peritoneal fluid in the immune balance of patients with endometriosis, showing an increase in the cytokines IL-2, IL-6 and TGF-β, an increase in amyloid protein A (SAA), as well as an increase in CD4(+)CD25(high)Foxp3(+) cells, and a decrease in ICOS+Treg (T regulatory cells) and CD45RO+Treg cells in patients with the disease [10,11,14].

This inflammatory process found in patients with endometriosis has been described because it is possibly related to the presence of microorganisms. Several authors have shown, for example, an increase in the cytokine IL-1β in patients with endometriosis and they correlate this increase with the activation of inflammasomes by stimulating microorganisms, and also correlate the participation of this event in the pathogenesis of the disease [17,18,19].

Several other studies shown the presence of microorganisms in menstrual blood, peritoneal fluid and vagino-uterine tract [20,21,22,23,24], but no work so far has described the presence of microorganisms in endometriotic lesions, and their correlation with microorganisms found in vaginal secretion and eutopic endometrium.

In this context, the present project aimed to investigate, by using high-throughput DNA sequencing, the microbiome profile present in vaginal fluid, endometrium and deep endometriotic lesions of women with endometriosis in comparison to microbiome profile found in vaginal fluid and endometrium of women without the disease. As there is a lack of understanding of the relationship between them, identifying endometriosis-associated microbiome profiles could help the understanding of pathogenesis and eventually could lead to the development of a noninvasive test for endometriosis.

## 2. Materials and Methods

### 2.1. Sample Collection

This case-control study was conducted as a pilot investigation at Hospital Israelita Albert Einstein (HIAE), São Paulo, Brazil, and the protocol was approved on December, 2017 by the Research Ethics Committee of Hospital Israelita Albert Einstein (project number 80280317.5.0000.0071); all patients provided written informed consent.

Twenty-one patients were included in the study according to the inclusion and exclusion criteria outlined below. Patients were assigned to the endometriosis group after lesion(s) identification by laparoscopic surgery, and further confirmation by histopathology analysis. Just women showing deep endometriosis were included. The control group consisted of women who underwent laparoscopic surgery for benign gynecologic diseases or elective tubal ligation in which peritoneal cavity inspection confirmed absence of endometriosis.

Women aged 18–50 years presenting eumenorrheic cycles were included in the study, whether or not under hormonal treatment. Women previously diagnosed with autoimmune, inflammatory and/or neoplastic disease, and who had used antibiotics/antimycotics in the 30 days prior to samples’ collection were excluded.

Eutopic endometrium and endometriotic lesion tissue samples were collected by curettage and laparoscopic surgery, respectively. All samples were collected at the operative room to minimize contamination and every care was taken to prevent the swab from touching other tissues adjacent to the target site, not having contact with blood, or any other instrument. Tissue samples were immediately frozen in liquid nitrogen and maintained at −80 °C. Vaginal fluid was sampled with sterile nylon flock swabs (Copan, Murrieta, CA, USA), also did not touched other sites and it was maintained in a microbiome transport solution (BiomeHub, Florianópolis, SC, Brazil) which allows a stable transport at ambient temperatures.

### 2.2. DNA Extraction and 16S rRNA Amplicon Sequencing

DNA from tissue samples was extracted using the DNeasy Power Soil Kit (QIAGEN). Processing of all samples was carried out under sterile conditions. The tissue fragments were initially minced and submitted to mechanical disruption; the DNA extraction followed the manufacturer’s protocol. Vaginal fluid samples in transport solution were extracted using the QIAamp DNA Blood Mini Kit (QIAGEN) according to the manufacturer instructions. Negative control samples were included for the extraction procedures, to evaluate kit reagent DNA background and possible process contaminations.

Preparation of an amplicon sequencing library for bacteria was performed using the V3-V4 16S rRNA gene primers 341F (CCTACGGGRSGCAGCAG) [25] and 806R (GGACTACHVGGGTWTCTAAT) [26], under the following conditions: the first PCR primers contained the Illumina sequences based on TruSeq structure adapter (Illumina, San Diego, CA, USA), allowing the second PCR with indexing sequences. The PCR reactions were always carried out in triplicates using Platinum Taq (Invitrogen, Waltham, MA, USA) with the conditions: 95 °C for 5 min, 25 cycles of 95 °C for 45 s, 55 °C for 30 s and 72 °C for 45s and a final extension of 72 °C for 2 min for PCR 1. In PCR 2 the conditions were 95 °C for 5 min, 10 cycles of 95 °C for 45s, 66 °C for 30 s and 72 °C for 45 s and a final extension of 72 °C for 2 min. Negative control reactions were included to access possible PCR reagent contaminations. The final PCR reactions were cleaned up using AMPureXP beads (Beckman Coulter, Brea, CA, USA) and samples were pooled in the sequencing libraries for quantification. The DNA concentration of the pool amplicon was estimated with Picogreen dsDNA assays (Invitrogen, Waltham, MA, USA), and then the pooled libraries were diluted for accurate qPCR quantification using a KAPA Library Quantification Kit for Illumina platforms (KAPA Biosystems, Woburn, MA, USA). The library pool was adjusted to a final concentration of 11.5 pM and sequenced in a MiSeq system, using the standard Illumina primers provided in the kit. A single-end 300nt run was performed using a V2 × 300 sequencing kit. Original sequencing data are available at NCBI BioProject PRJNA546137.

### 2.3. Microbiome Profile Evaluation Through Bioinformatics Analysis

After the amplicon sequencing, the EncodeTools Metabarcode Pipeline (BiomeHub, Florianópolis, SC, Brazil) was used [27]. In this pipeline, the sequenced reads were quality filtered and primers trimmed resulting in a read of 283pb. Sequence reads smaller than expected, with remaining adapter sequences or more than one mismatch in the primer were excluded. All the reads that passed this quality assessment were clustered into 100% identity oligotypes and analyzed with Deblur [28] and the VSEARCH [29] packages to remove possible erroneous and chimeric reads. Oligotypes below a frequency of 0.2% in the samples were removed. Additionally, a negative control filter was implemented. If any oligotype was observed in the negative controls, it was checked against the samples and removed from the results.

After all, the oligotypes were used for taxonomical assignment with the BLAST [30] tool against a reference genome database constructed with NCBI and *in-house* bacterial genome sequences. Taxonomy was assigned to each oligotype using a lowest common ancestor (LCA) algorithm. If more than one bacterial reference can be assigned to the same oligotype with equivalent similarity and coverage metrics, the EncodeTools Metabarcode Taxonomy Assignment algorithm leads the taxonomy to the lowest level of possible unambiguous resolution (e.g., genus, family, kingdom) according with the similarity thresholds previously stablished [31].

Microbiome data comparison and diversity analysis were conducted inside the R statistical environment (R version 3.6.0). The Phyloseq R package [32] was used for alpha diversity analysis in the plot_richness function. Raw amplicon sequences were used to construct phylogenetic trees using FastTree 2.1 [33] and these were considered to calculate weighted UniFrac distances [34]. Beta diversity analyses used a proportion-normalized abundances [35,36] to calculate Bray–Curtis dissimilarity and weighted UniFrac using the Phyloseq’s distance function. Nonparametric tests, including the Kruskall-Wallis and Wilcoxon tests were used as implemented in base R and in coin R package [37].

DESEq2 R package was used to identify differentially abundant genera with the Generalized Linear Model implemented [38]. The obtained *p*-values were corrected according to Benjamini and Hochberg procedure [39]. Values were reported as fold-changes in the log2 scale (log2 FC).

Graphical visualizations for the analysis in the boxplots, principal coordinates analysis (PCoA) and heatmap were generated with the ggplot2 R package [40].

## 3. Results

Twenty-one patients were included in this study, eleven in the control group and ten in endometriotic group. A total of 47 samples were collected and processed for the evaluation of their bacterial profile: 21 samples of vaginal fluid, 18 eutopic endometrium and 8 endometriotic lesion samples.

Considering all collected samples, we were able to identify 51 different bacterial genera, in a total of 414,787.00 reads, with an average of 8465.04 reads per sample.

**Figure 1:**
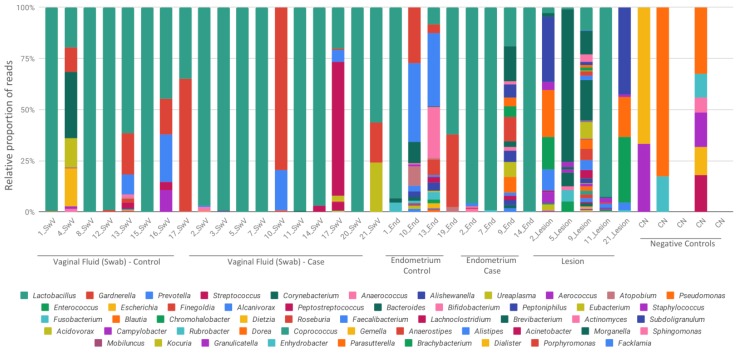
Relative abundance of bacterial profiles. Bacterial composition of each sample is reported by color bars relative to a 100% scale. Results are presented by the taxonomic rank of genus. SwV—vaginal fluid (Swab), End—endometrium, CN—negative controls.

Microbiome sequencing from vaginal fluid, eutopic endometrium and endometriotic lesions resulted in similar microorganism profiles, which were composed most abundantly by the bacterial genus *Lactobacillus*, *Gardnerella*, *Streptococcus* and *Prevotella* (Figure 1).

**Figure 2:**
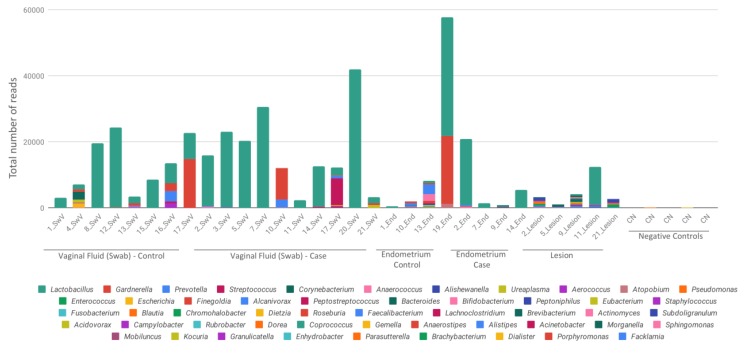
Total reads sequenced for each sample. Bacterial composition of each sample is reported by different color bars and scaled as the total number of reads for each sample. Results are presented by the taxonomic rank of genus. SwV—vaginal fluid (Swab), End—endometrium, CN—negative controls.

Eutopic endometrium, as well as endometriotic lesion samples, had lower amount of detected relative reads in comparison to vaginal fluid samples (Figure 2), on the other hand, these samples showed less diversity, with which showed a marked predominance of *Lactobacillus*.

We found that endometriotic lesion samples had the most microorganism diversity which included *Lactobacillus*, *Enterococcus*, *Gardnerella*, *Pseudomonas*, *Alishewanella*, *Ureaplasma* and *Aerococcus* (Figure 1).

Negative controls (CN) were included in Figure 1 and Figure 2 to demonstrate the lower and different number of reads obtained from kits and reagents background. An average of 66 reads per control was recovered, with a minimum of 45 and a maximum of 204. Negative controls have a totally different bacterial profile from the samples, which demonstrates that there was no contamination of the reagents, and that the presence of microorganisms comes from the studied samples.

**Figure 3:**
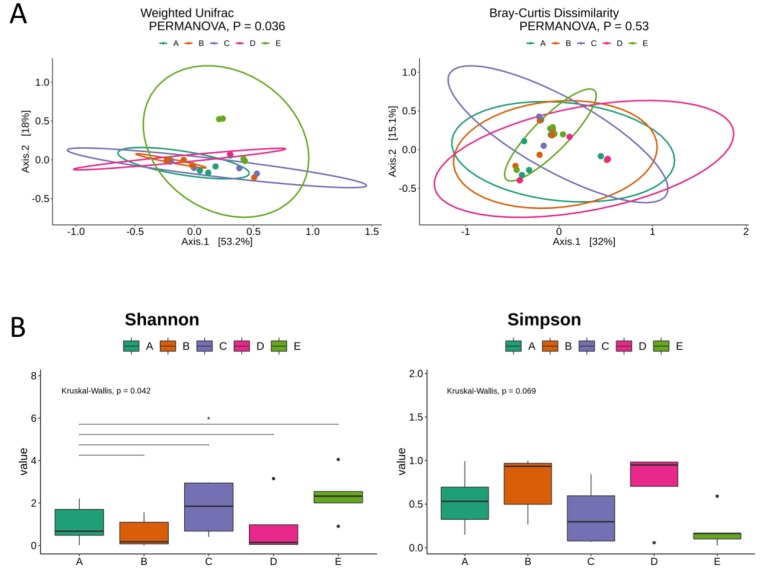
Alpha and beta-diversity analysis. (**A**) Principal coordinates analysis (PCoA) made with weighted UniFrac distances and Bray‐Curtis dissimilarity. Sample groups are represented by the letters: A (dark green)-control swab from vaginal fluid; B (orange)-case swab from vaginal fluid; C (purple)-control endometrial tissue; D (pink)-case endometrial tissue; and **E** (light green)-lesion. (**B**) Alpha diversity indexes of Shannon and Simpson are sowed in the boxplots for each collection site group of samples (A, B, C, D and E). Kruskall‐Wallis and Wilcoxon tests are showed for group and paired groups comparison, respectively (represented by solid lined above boxplots, only significative comparisons were shown and marked with an *). Black solid points represent outlier samples.

A slightly significant difference (*p* = 0.036) among the bacterial composition of lesions (E) compared to other samples was observed when beta-diversity analysis, with weighted UniFrac distances were used, but no differences among samples were observed in Bray‐Curtis dissimilarity (Figure 3A).

Already when alpha-diversity analysis considering Shannon and Simpson indices was used, we observed highly variable results, but similar diversity levels with the other samples (Figure 3B), despite the tendency to a lower alpha-diversity in endometriotic lesions compared to control vaginal fluid samples.

**Figure 4:**
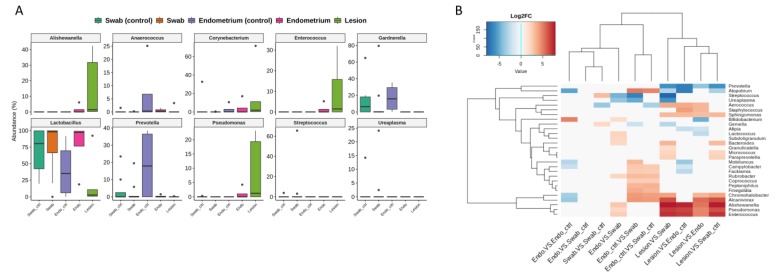
Relative and differential abundances. (**A**) Boxplots represent the most abundant genera detected and their distribution in the samples. Swab_ctrl—control swab from vaginal fluid; Swab—case swab from vaginal fluid; Endo_ctrl—control endometrial tissue; Endo—case endometrial tissue; lesion—samples from endometriosis lesion. Black solid points represent outlier samples for each group. (**B**) Differential abundance heatmap comparing all the collection sites from case and control samples. Results are showed as the log2FC for the differentially detected genera. Most abundant genera are showed as red values considering the first sample category in the bottom legend.

The most abundant genera detected in the collection sites were plotted (Figure 4A) and it was possible to observe some patterns like the predominance of *Lactobacillus* in vaginal fluid and endometrial samples. The high abundance of *Gardnerella* and *Prevotella* could be observed in the control samples of vaginal fluid and endometrium, while endometriotic lesions showed a higher prevalence of *Alishewanella*, *Enterococcus* and *Pseudomonas*. This bacterial profile of lesions was also detected in DESeq2 differential abundance analysis, with a significant result (Figure 4B).

## 4. Discussion

In this study, we report the microbiome profile from deep endometriotic lesions in Brazilian women.

We detected the presence of microorganisms of the genus *Lactobacillus*, *Enterococcus*, *Gardnerella*, *Pseudomonas*, *Alishewanella*, *Ureaplasma* and *Aerococcus* in deep endometriotic lesions of fifty percent of our endometriosis patient group.

When we compared the microorganism profile from lesions with that found in vaginal fluid and eutopic endometrium of patients with endometriosis, slightly significant differences were found, with a major diversity seen in lesions. Beside this difference, this microbiome data did not reveal a strict bacterial profile specific to the collection sites.

Similar profile between samples derived from 14 women with endometriosis and 14 healthy controls also was observed in a study conduct by Ata et al. [24]. Despite overall similar vaginal, cervical and intestinal microbiota composition between endometriosis and control groups, the authors observed differences at the genus level, with an absence of *Atopobium* in the vaginal and cervical microbiota of the endometriosis group, and enhanced presence of *Gardnerella*, *Streptococcus*, *Escherichia*, *Shigella*, and *Ureaplasma*.

Our results reflect similar profiles of microorganisms as also those obtained by Chen et al. [23] for vaginal and endometrial samples, where the samples collected from endometrium tissue showed a transition profile from the vagina to the upper reproductive tract without sufficient differentiation from other sites of the female reproductive tract. However, the authors still found a change in the microbiota in the peritoneal fluid of women with endometriosis. This alteration found in the peritoneal fluid reinforces our hypothesis that there is an involvement of microorganisms and the immune system in the process of establishment and maintenance of lesions.

Our group investigated the expression of toll-like receptors (TLRs), in T reg cells isolated from peritoneal fluid from patients with and without endometriosis, and we found that there is a change in the activation of TLRs receptors in women with endometriosis, where TLR 1, 2, 3, 4, 5, 6, 7 and 8 were expressed in regulatory T cells isolated from peritoneal fluid from women with endometriosis while only TLR1 and TLR2 receptors were expressed in women without endometriosis [41].

These findings are in line with the findings of this study, which shows that endometriotic lesions have a greater diversity of microorganisms, which consequently cause Treg cells to express a greater number of toll-type receptors, and that there is a relationship between the presence of microorganisms and the inflammatory process.

A retrospective study involving 141.460 patients, out of which 28,292 presented PID (pelvic inflammatory disease) and 113,168 were healthy, showed the correlation between microorganisms and inflammation, suggesting that PID patients were at high risk (three times more) for developing endometriosis than those without PID, suggesting that such bacterial transport in women with PID could facilitate contamination of the upper reproductive tract and pelvic cavity, leading to endometriosis [42]. PID is characterized as a disease caused by the passage of pathogenic bacteria from the vagina to the uterus, fallopian tubes and ovaries.

Present in the plasma membrane or inside vesicles, TLRs are the first receptors to recognize molecular signals of pathogen-associated patterns (PAMPs), triggering a cascade of cellular events. Next, receptors present in the cytosol are activated, via activator protein (AP1) and via interferon regulatory factors (IRFs). These receptors form cytosolic molecular complexes, called inflammasomes, activate caspase- 1 and the production of cytokines, such as IL-1β and IL-18 that induce pyroptosis and apoptosis [43,44,45,46,47]. This process involving TLR receptors, inflammasomes and cytokine production leads to the immune system’s response against a wide variety of microorganisms.

These would dampen the natural inflammatory process responsible for the elimination of the retro-flowing uterine endometrial cells during menstruation. As a result, the attachment of ectopic endometrial cells to the peritoneal surfaces would be facilitated. This hypothesis has also been brought forward by other studies related to this topic [48,49,50,51,52,53,54].

Our finding of microorganisms in the endometriotic lesions themselves is important as it may advance the hypothesis that one or all of these also ectopically found bacteria may be implicated in the maintenance and survival of the ectopic endometrial cells. The fact that they are also found in the uterine cavity and in the vagina would only be expected.

This was a preliminary evaluation of endometriotic lesions microbiome and there are limitations to the study. A new study with a larger number of patients has to be carried out to confirm the results observed in patients with endometriosis. Moreover, the relation between the presence of these microorganisms and their metabolites in the peritoneal fluid or in the endometriosis lesions should be explored, especially looking into regulation of inflammation and immune responses. As one of the functions of the peritoneal fluid is to remove pathogens the presence of microorganisms in the peritoneal cavity may indicate a poor in situ immune response, one of the possible causes of endometriosis [55].

## 5. Conclusions

In conclusion, this study revealed that deep endometriotic lesions present a diversity of bacteria as observed in a microbiome analysis using high-throughput DNA sequencing methods. We understand that this result may open new avenues to the study endometriosis and eventually could lead to the development of a noninvasive test for the disease.
